# Nest boxes increase reproductive output for Tree Swallows in a forest grassland matrix in central British Columbia

**DOI:** 10.1371/journal.pone.0204226

**Published:** 2018-10-10

**Authors:** Andrea R. Norris, Kathryn E. H. Aitken, Kathy Martin, Stanley Pokorny

**Affiliations:** 1 Department of Forest and Conservation Sciences, University of British Columbia, Vancouver, British Columbia, Canada; 2 School of Science, Yukon College, Whitehorse, Yukon, Canada; 3 Science and Technology Branch, Environment and Climate Change Canada, Delta, British Columbia, Canada; Cornell University, UNITED STATES

## Abstract

Secondary cavity-nesting birds depend on tree cavities for nesting and roosting, but many studies of these birds are conducted using nest boxes. Implementation of effective conservation strategies for cavity-nesting species such as nest-site supplementation requires careful comparisons of fecundity and other vital rates for birds using both natural and artificial nest site types. We compared breeding phenology, clutch and brood sizes, and fledging success of Tree Swallows (*Tachycineta bicolor*) nesting in tree cavities and nest boxes during 2001–2003 in British Columbia, Canada. Swallows using nest boxes initiated egg-laying and hatched young at approximately the same time as those in tree cavities (2 June, 23 June, respectively). Female Tree Swallows in boxes laid larger clutches (5.9 ± 0.9 eggs, *N* = 76) than those in tree cavities (4.2 ± 1.6 eggs, *N* = 67). The mean number of nestlings hatched was greater in nest boxes (5.2 ± 1.1 nestlings, *N* = 67) than in tree cavities (2.6 ± 2.0 nestlings, *N* = 58). Pairs in boxes were over twice as successful in producing fledglings (93.4%; 57 of 61 pairs fledged > 1 young) than those in tree cavities (35.8%; 19 of 53 pairs). Of those successful nests, pairs nesting in boxes fledged 5.1 ± 1.1 young (N = 57), whereas those in tree cavities fledged 3.5 ± 1.2 young (N = 18). Because cavities in nest boxes averaged 60% larger in volume and 1.8 cm wider internally than tree cavities, we suggest that increased reproductive output was correlated with boxes enabling a larger clutch size. In previous research, we found that Tree Swallows were a poor competitor with other cavity-nesting passerines for tree cavities. The addition of nest boxes may serve as an effective way to supplement local reproduction for secondary cavity-nesting bird populations by reducing competition for limited nest sites. This is especially true in regions where the availability of natural nesting sites is highly variable, and where species compete with many other cavity-nesting passerines using a similar ecological niche and nesting cavities.

## Introduction

Tree Swallow (*Tachycineta bicolor*) populations, like many other aerial insectivores in North America, have shown continent-wide declines since the 1980s [1,2]. Providing nest boxes is one popular management technique promoted for the recovery of species such as Tree Swallows that require cavities for nesting [3]. However, nest boxes can become ecological traps when a cue such as nest size leads cavity-nesters to prefer boxes over tree cavities despite boxes being located in areas of higher predation risk and/or lower food availability (and subsequently lead to lower reproductive success and/or survival [4]). Thus, local increases in breeding densities after the addition of nest boxes and other artificial cavities, as shown in many species [5–9], might be an insufficient metric in the assessment of this technique in promoting population recovery [10].

Comparisons of the breeding biology of birds nesting in tree cavities and nest boxes has revealed that pairs in nest boxes may initiate clutches earlier [11]and have larger clutches [11–13], experience lower predation rates [11,12,14,15], and produce more fledglings [6–9,11,15–18]. In contrast, other studies have revealed that birds nesting in tree cavities and nest boxes did not differ in clutch initiation dates [11,13,17,19], or clutch sizes [11,17,19], and that nest predation rates were greater in boxes than in natural cavities in some years [11,13,20]. The risk that nest-boxes might be ineffective at improving reproductive success, or worse, further exacerbate population declines must be explored in order to test the effectiveness of nest-site supplementation for the implementation of meaningful conservation programs.

A number of mechanisms may explain differences in reproductive output between birds nesting in nest boxes and tree cavities. Studies reporting differences in the initiation of egg laying between nesting contexts have suggested that, 1) abundant nest boxes placed in areas where competition for tree cavities is high may allow pairs to settle earlier in boxes [11], and/or 2) a greater number of older females settle in nest boxes and initiate laying earlier [11–13]. One possible explanation for the discrepancies in clutch size between nest box and tree cavity nests is that females adjust clutch sizes based on the dimensions of the cavities [13,21]to decrease the negative effects of crowding on nestlings [22]. An increase in clutch size with increasing nest cavity size has been reported for some bird species such as Great Tits (*Parus major*), Pied and Collared Flycatchers (*Ficedula spp*.; [23,24]), and Tree Swallows [24,25], but not for other species such as European Starlings (*Sturnus vulgaris*), Eastern Bluebirds (*Sialia sialis*), American Kestrels (*Falco sparverius*), and Northern Flickers (*Colaptes auratus*; [21,22,26,27]).

Ectoparasites can reduce the mass, growth rate, and survival of nestlings of cavity-nesting species [28–32]. Even though removing nest material between breeding seasons is a common practice in nest box studies, it is rarely, if ever, reported [33,34]. Nest boxes containing old nests that are not removed between breeding seasons can have a greater number of ectoparasites (bird fleas; *Ceratophyllus idius*) than cleaned boxes, more closely mimicking the ectoparasite loads found in tree cavities [34]. Therefore, many studies reporting greater reproductive output in nest boxes may be an artefact of reduced ectoparasite loads in clean boxes [33].

Predation rates could vary between boxes and tree cavities given that boxes are often lower to the ground, placed in exposed locations, and located in greater densities than natural tree cavities, thus making boxes more vulnerable to predators. In continental Europe, nest predation rates by Greater Spotted Woodpeckers (*Dendrocopus major*) were greater in boxes located closer to the ground compared to tree cavities for European Starlings, Eurasian Blue Tits (*Cyanistes caeruleus*) and Marsh Tits (*Poecile palustris*) [12,14]. However, these same studies and others in the UK reported the opposite result of lower woodpecker predation rates in boxes for tits and Pied Flycatchers (*Ficedula hypoleuca*), relative to tree cavities [12,14,15]. As a result of higher predation rates in nest boxes, researchers often modify boxes to limit predation [35,36]. A recent analysis of nest fate data in nest boxes across the United States and Canada found that nest survival was improved with the presence of predator guards for most species examined, including Tree Swallows [36]. The presence of predator guards, therefore, might explain some discrepancies in reproductive success between birds nesting in boxes and tree cavities. Thus, differences in reproductive variables between nest types and their suggested mechanisms are often variable both across and within species. Further, meta-analyses are complicated by inconsistencies in reporting whether nest boxes are cleaned and/or have been modified to limit nest predation.

Much of what we know about how nest characteristics influence the breeding biology of secondary cavity-nesting passerines comes from studies of Tree Swallows in nest boxes [3,37–44]. Researchers studying Tree Swallows in Ontario, Canada found that birds using nest boxes tended to be older females that initiated clutches earlier and had larger clutches than those nesting in tree cavities [13]; however, in that study, nestling survival rates varied with year and the proportion of nests fledging young did not vary with nest site type. Further, this study reported on a limited sample size of reproductive variables in tree cavities that were located up to only 3 m above standing water despite nest heights ranging up to > 9 m above water [13,24]. In another study of Tree Swallows in interior British Columbia, Canada, researchers found that laying date and clutch size did not differ between pairs using nest boxes and tree cavities, but results were inconclusive as a result of a very small sample size [45]. Thus there is a need to examine the question of the influence of nest site type in a setting where the full range of natural tree cavities can be compared with uncleaned nest boxes (i.e., nest contents not removed between breeding seasons) that do not have predator guards, and where both nest site types are in close proximity to food.

During three years (2001–2003) of our long-term study on cavity-nesting birds [46], we examined the fecundity of Tree Swallows nesting in cavities (*N* = 123) and uncleaned nest boxes that lacked predator guards (*N* = 78) in the same habitat types but with experimentally manipulated supplies of nest-sites in central British Columbia. We hypothesized that annual reproductive output (breeding phenology, clutch and brood sizes, and fledging success) of Tree Swallows differed between pairs using tree cavities and nest boxes.

## Materials and methods

We conducted fieldwork from May through July 2001–2003 in mixed coniferous-deciduous forests within the Interior Douglas-fir biogeoclimatic zone of central British Columbia, Canada [47]. Predominant tree species were lodgepole pine (*Pinus contorta*; 41%), Douglas-fir (*Pseudotsuga menziesii* var. *glauca*; 28%), trembling aspen (*Populus tremuloides*; 16%), and white and hybrid spruce (*Picea glauca x engelmannii*; 15%, [48]). Our study sites were located near Riske Creek, 40 km west of the City of Williams Lake, and near Knife Creek, 20–40 km east of Williams Lake (52°14’N, 122°12’W). Our 20 sampling sites (most 15–32 ha in size) varied in composition from continuous forest to five sites that consisted of forest groves (0.2–5 ha). Small gaps in continuous forest stands comprised grasslands, shallow ponds and small clearcuts [46]. Forest groves were spaced a mean of 84 m (range 16–222 m) from the nearest grove or forest within a grassland matrix [7]. Suitable tree cavities (those used at least once for nesting by any cavity-nesting species, which we monitored as part of a concurrent long-term monitoring study of cavity-nesting vertebrates; [7,9,46,48–50]) were a mean of 22.7 m (range 0–650 m) from the forest edge, but cavities used by Tree Swallow for nesting were a mean of 10.5 m (range 0–50 m) from the edge (K. Martin unpublished data). We placed nest boxes 3 m to 30 m apart along fence lines and at forest edges 500 m to 1 km from our Riske Creek tree cavity study sites. Of 100 boxes monitored from 2001 to 2003, 90 were present for at least two years prior to our study, with nine added in 2002, and another one added in 2003. We monitored Tree Swallow nests in boxes at six sites and in tree cavities at 19 of the 20 sites.

Boxes were rectangular, top-opening, and constructed of 1.27-cm-thick plywood. For nest boxes, entrances were circular with a mean diameter of 5.2 ± 1.9 (SD) cm, mean entrance height above ground was 1.6 ± 0.6 (SD) m (*N* = 60), mean internal depth was 14.6 ± 3.1 cm (*N* = 74 boxes), mean internal width was 13.3 ± 1.6 cm, and mean volume was 2582.6 ± 8.0 cm^3^ (*N* = 76). The dimensions of nest boxes allowed access for swallows, bluebirds, chickadees, squirrels, woodrats, chipmunks, and mice, only, and prevented use by starlings, flickers, and kestrels. Nest boxes were not modified to prevent nest predation (i.e., predator guards were not used on any boxes). For nest cavities (1995–2002, mean entrance height above ground was 4.3 ± 2.5 m (*N* = 190), mean internal depth was 15.1 ± 8.2 cm (*N* = 92), mean internal width was 11.5 ± 4.2 cm, and mean volume was 1568.4 ± 113.6 cm^3^ (*N* = 107; [36]). Additional details about our study area are provided in [48–50].

### Nest monitoring

We conducted systematic nest-searches by checking tree cavities used in previous years and all nest-boxes approximately every 4 days, and we monitored nests in tree cavities (excavated or natural decay-formed holes) and in boxes. We visually inspected nest boxes and tree cavities using a mirror and flashlight. Nest boxes were generally within reach of the ground, but inspection of tree cavities usually required the use of a ladder. Throughout the study, we recorded and did not remove all old and new nest materials and contents including unhatched eggs and dead nestlings. For each nest, we recorded the date the first egg was laid (date of first egg), the maximum number of eggs observed during incubation (where the same number of eggs was observed on ≥ 2 visits; clutch size), hatch date, and the maximum number of eggs to hatch (brood size) and the maximum number of nestlings to fledge. To determine date of first egg at nests found after the first egg was laid, we assumed that one egg was laid per day and counted backwards from the date that the final egg was laid at nests found during laying. We determined hatch date as the first day we observed at least one chick hatched, or we estimated hatch date from chick age based on characteristics of the nestlings [3,51,52]. To quantify date of first egg and hatch date, we used the number of days since 1 January (Julian day). When nestlings were approximately 10 days old (post-hatching), we weighed and marked them with aluminum bands, and then placed them back in the cavity or box. We captured some adults at the nest by placing a net over the cavity or box entrance, and measured, marked with aluminum and colour bands, and then released them immediately. However, we captured an insufficient number of adults to assess how age and experience might have influenced reproduction in this study. We monitored nest boxes and cavities until they were empty (chicks fledged or nests failed). Successful nests were those where we observed fledging by at least one chick, or at least one chick was in the nest at least 18 days after hatching (with no evidence of predation after the nest was empty), the minimum number of days required for Tree Swallow nestlings to fledge [3]. Nest depredation was identified by the absence of eggs or nestlings before the minimum number of days required for fledging and/or the presence of a predatory species inside the cavity, or teeth marks in the wood around the cavity entrance, and/or mammal fur or feces inside the cavity entrance or on the cavity floor. All field data were collected under annually renewed permits under the University of British Columbia Animal Care Protocol, and Environment and Climate Change Canada’s Scientific permit and banding permit number 10365.

### Statistical analyses

We used five generalized linear mixed effects models to compare the fecundity characteristics (date of first egg, clutch size, brood size, hatch day, and fledging success) of nests in tree cavities to those in nest boxes. For each model except date of first egg laid, the fixed effects were nest type (natural tree cavity or nest box) and year. In the model with the response variable, date of first egg laid, we included year as a random effect to divide the error terms into year versus measures within year thereby partially accounting for correlations of repeated measures within years. We matched nests in cavities and boxes with the same date of first egg laid, and assigned a unique identifier, TempID. To account for temporal variation in fecundity variables while comparing reproductive output between nest types (cavities vs. boxes), we included TempID as a random effect in all models except where date of first egg laid was the response variable.

For both clutch and brood size, data were non-normal integers, and the residual deviance of Poisson models was not equal to the degrees of freedom (DF), so we specified a quasi-Poisson distribution for both variables. For date of first egg and hatch day, we used normal distributions, and visually examined plots of residuals to ensure that errors were homoscedastic and that the models fit the data. For fledging success, we specified a binomial distribution (fledged = 1, failed = 0), and used a logit link function. Because nests found at different nesting stages can bias nest success estimates [53], we applied a logistic-exposure adjustment to the binomial distribution in the fledging success model. Each observation was weighted by the inverse of the number of days that the nest was observed, thus providing estimates for fledging success without assuming when nest loss occurred [54]. We used penalized quasi-likelihood to generate parameter estimates for clutch size, brood size, and fledging success, and restricted maximum likelihood for date of first egg and hatch day, with the functions glmmPQL and lme, respectively, in the program R version 2.12.1 [55,56].

## Results

We found 112 Tree Swallow nests in tree cavities (33 in 2001, 36 in 2002, and 43 in 2003) and 78 in nest boxes (15 in 2001, 42 in 2002, and 21 in 2003). We found that 95% of box nests (*N* = 78) were in or near forest groves whereas only 66% of tree-cavity nests (*N* = 112) were in these sites.

**Fig 1:**
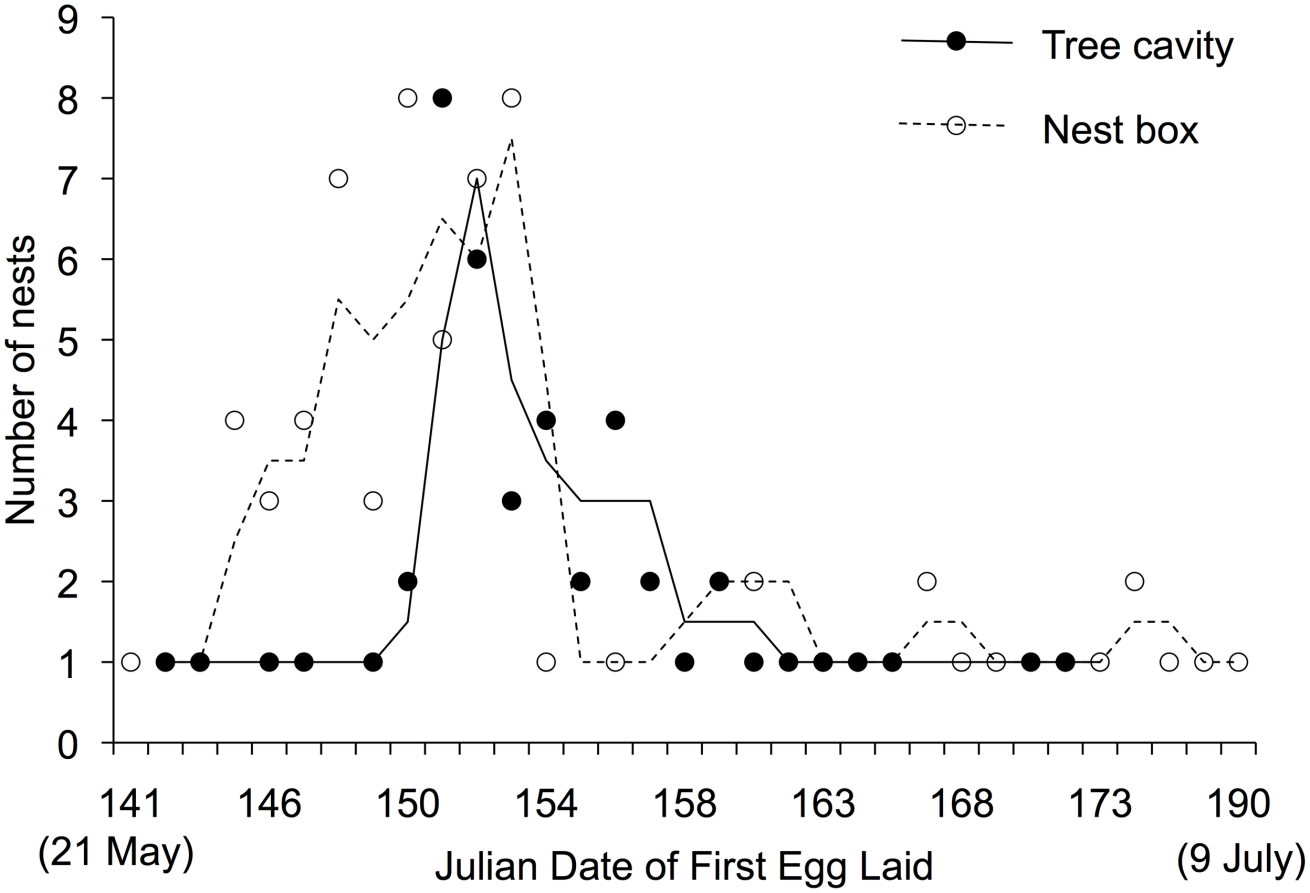
Julian date of first egg laid (DFE) by Tree Swallows nesting in tree cavities (*N* = 46) and nest boxes (*N* = 72) from 2001 to 2003 in interior British Columbia, Canada. Lines indicate a moving average (moving mean number of nests across date of first egg) for each nest type. This is calculated using the number of nests across two times: the DFE where the data point corresponds to the line and one data point prior.

**Fig 2:**
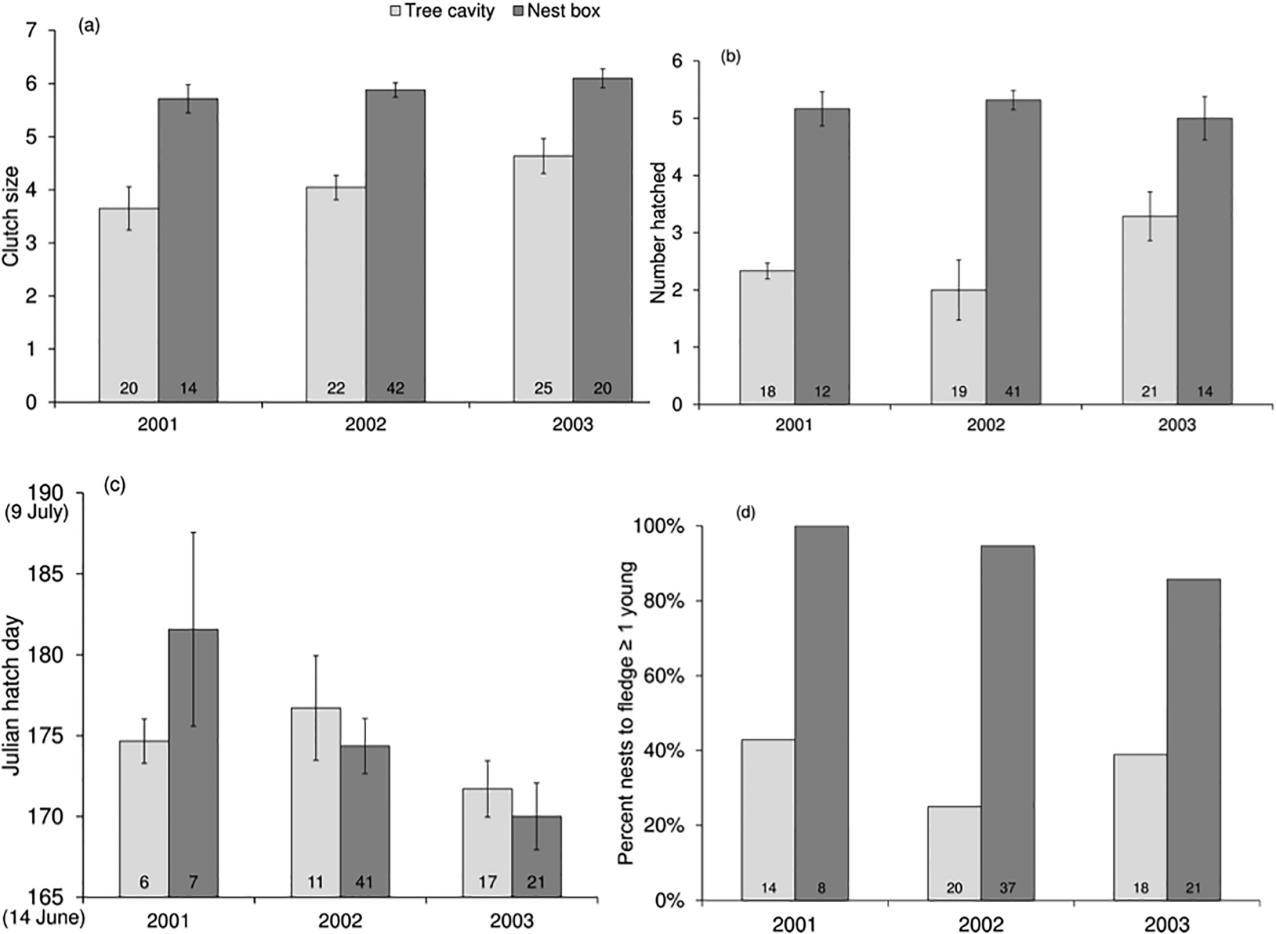
Reproductive output for Tree Swallows nesting in tree cavities and nest boxes, in interior British Columbia, Canada. (a) Mean number of eggs laid (clutch size) (b) mean number of chicks hatched, (c) mean hatch day (Julian hatch day), and (d) percent of nests to fledge ≥ 1 young. Sample size indicated at the base of each column. Error bars indicate standard errors of the mean.

Female Tree Swallows in nest boxes laid larger clutches (5.9 ± 0.9 eggs, *N* = 76 nests) than those using tree cavities (4.2 ± 1.6 eggs, *N* = 67 nests; Table 1, Fig 1). Variation in fecundity (number of eggs laid and hatched, and percent of young to fledge) was greater in tree cavities than in nest boxes (Table 1, Fig 2). However, mean date of first egg laid (2 June) nor mean hatch day (23 June) differed between nests in boxes and tree cavities (Table 1, Fig 2c). In tree cavities, clutch size was marginally negatively correlated with cavity entrance area (t = -2.05, df = 22, p-value = 0.05), and not correlated with the internal vertical depth (t = -0.18, df = 22, p-value = 0.85), nor the internal horizontal depth (t = 0.45, df = 22, p-value = 0.65).

**Table 1:** Generalized linear mixed-effects model parameter estimates, standard errors (SE), degrees of freedom (DF), test statistics (*t*-value), and *P* values for fecundity variables of Tree Swallows nesting in nest boxes versus tree cavities (Natural) and whether fecundity variables observed in 2001 differed from those in 2002 and 2003 (Year 2002, Year 2003). The signs of parameter estimates indicate whether clutches were laid/hatched earlier (-) or later (), whether clutches/broods were smaller (-) or larger (), and whether nests fledged at least one young () or failed (-) more frequently in tree cavities compared to nests in nest boxes. We included year as a random effect in the model, Date of first egg laid. Nests with the same date of first egg laid were grouped together and given a unique identification number, TempID, which was included as a random effect in all models except where date of first egg laid was the response variable.

Response variable	Fixed effect	Parameter estimate	SE	df	*t* value	*p* value
*Date first egg laid*	Intercept	152.75	1.67	107	91.28	**<0.01**
	Natural	1.93	1.54	107	1.26	0.21
*Clutch size*	Intercept	1.77	0.06	73	27.43	**<0.01**
	Natural	-0.30	0.05	73	-6.10	**<0.01**
	Year 2002	-0.01	0.07	73	-0.17	0.87
	Year 2003	0.05	0.07	73	0.73	0.47
*Number chicks hatched*	Intercept	1.68	0.10	71	16.24	**<0.01**
	Natural	-0.56	0.08	71	-6.61	**<0.01**
	Year 2002	-0.03	0.11	71	-0.24	0.81
	Year 2003	-0.01	0.12	71	-0.05	0.96
*Hatch day*	Intercept	180.53	2.61	65	69.17	**<0.01**
	Natural	-0.73	1.07	65	-0.68	0.50
	Year 2002	-2.97	1.70	65	-1.75	0.09
	Year 2003	-1.77	1.70	65	-1.04	0.30
*Fledging success*	Intercept	7.08	1.15	68	6.15	**<0.01**
	Natural	-2.77	0.77	68	-3.62	**<0.01**
	Year 2002	-0.89	1.05	68	-0.85	0.40
	Year 2003	-0.94	1.02	68	-0.92	0.36

Mean brood sizes were significantly larger in nest boxes (5.2 ± 1.1 nestlings, *N* = 67 nests) than in tree cavities (2.6 ± 2.0 nestlings, *N* = 58 nests; Table 1). Again, annual variation in brood size was greater in tree cavities than in nest boxes (Table 1, Fig 2b). The nestlings in the boxes weighed 21.4 ± 2.6g approximately 10 days after hatching, and nestlings in cavities were 22.0 ± 1.9g at approximately 10 days old.

Overall, Tree Swallow nests in boxes were over twice as successful in producing fledglings (93.4%; 57 of 61 pairs fledged ≥ 1 young) than those in tree cavities (35.8%; 19 of 53 pairs fledged ≥ 1 young; Table 1). Of the pairs to fledge ≥ 1 young (i.e., nest fate was successful), those in boxes fledged 5.1 ± 1.1 young (*N* = 57), and those in tree cavities fledged 3.5 ± 1.2 young (*N* = 17). For cases in which we could confirm nest failure in tree cavities, 56% (19 of 34 nests) were depredated. We could confirm nest failure in only five nest boxes (of 78 nests), two of which were depredated. We found little annual variation in fledging success across nest site types (Table 1, Fig 2d).

## Discussion

Our result that reproductive output was greater in nest boxes than tree cavities suggests that nest boxes offer a reproductive advantage even when no attempts are made to prevent ectoparasitism or nest predation. We suggest, therefore, that installing nest boxes could be an effective strategy for bolstering populations of Tree Swallows.

Interspecific competition for tree cavities that leads to later settlement may help explain why Tree Swallow clutches in cavities were smaller than those in nest boxes. Researchers in Ontario suggested that interspecific competition from earlier breeding European Starlings, the most abundant competitor, led to later and smaller Tree Swallow clutches in tree cavities [13]. In that study, the high densities of Tree Swallows (6.6 pairs/ha) occupying the tree cavity sites likely contributed to high competition for cavities [13]. Likewise, during our study, Tree Swallow pairs (N = 112 pairs) had to compete for breeding sites with more dominant species of comparable abundance (84 Mountain Bluebird; *Sialia currucoides* pairs, 114 European Starling pairs and 130 Northern Flicker pairs; K. Martin unpublished data) that initiated nesting early [57]. In a separate study at our study site, Tree Swallows proved to be an inferior nest-site competitor, initiating clutches 7–14 days later at cavities in years and areas with breeding European Starlings and Mountain Bluebirds present. Additionally, changes in nest-site supply occurred with a separate study in which cavities were removed and added at our study area during 2002–2004 [7,9]. In this other study, cavities were removed (blocked) at some sites [7] and added at other sites in 2002 and 2003 [9]. At sites where cavities were removed, we suspect that Tree Swallows used cavities of lesser quality resulting in small clutches but exhibited no site-level changes in population densities [7]. At sites where nests were added, Tree Swallows used only a very small proportion of the new nest sites and therefore likely still had to compete for cavities with the inflated densities of competitors [9]. Thus, the smaller clutches and broods in tree cavities were apparently correlated with an increase in competition for nest-sites, and swallows nesting in boxes were able to maintain larger clutches.

Tree Swallows preferentially selected and were more successful in smaller cavities produced by Red-naped Sapsuckers (*Sphyrapicus nuchalis*) whereas bluebirds and starlings preferred larger cavities excavated by Northern Flickers [49,57]. This may help to explain why our results differed from a similar study in the same region in British Columbia, which found no difference in reproductive output of Tree Swallows nesting in boxes and cavities [45]. The other British Columbia study [45] examined nests in boxes that were erected for ducks, which had large cavity entrances. In our study, nest boxes had much smaller cavity entrances, therefore swallows that used boxes avoided competition with starlings and flickers, and produced larger and more successful clutches and broods. Nest boxes were 60% larger in volume, and 1.8 cm wider internally than tree cavities used by Tree Swallows. Other researchers [13] found positive correlations between cavity floor area and clutch size and between cavity floor area and probability of fledging young of Tree Swallows. We found that clutch size marginally declined with increasing tree cavity entrance area, but found no evidence of a relationship with internal cavity size. Further, we were unable to examine the correlation between clutch size and nest box volume due to the lack of variation in nest box size. Future work to test the hypothesis that nest volume influences clutch size should involve supplementation of nest boxes with a wide range in internal volume. It is important to note, however, that cavity entrance should be kept small to restrict larger bodied competitors and predators.

We found a greater number of nests later in the season in boxes, particularly in 2001 (Figs 1 and 2c), and due to logistical constraints during the study we were unable to confirm nest success or failure in these later nests. Nonetheless, fledging success was consistently higher in nest boxes across all three years of our study, including the year before nest-site removal and supplementation (2001), suggesting that competition for tree cavities alone cannot explain higher nest success in boxes. Our results differ from another study on Great Crested Flycatchers (*Myiarchus crinitus*) in northern Florida that found high annual variation in nest success in cleaned nest boxes but consistent nest success in tree cavities, which they attributed to different nest predator species depredating the two nest types [20]. We found on multiple occasions that grass nests were built by small mammals inside cavities and boxes, indicating that nest predators frequently occupied both nest types. In our long-term study of the cavity-nesting community, the most abundant predators for nests in trees were red squirrels (*Tamiasciurus hudsonicus*; [58]). Others have shown nest predation rates of Tree Swallows to increase in cavities closer to the ground [24]. Relative to tree cavities, our nest boxes lacked predator guards, had the same range of potential nest predators, and were closer to the ground.

The presence of ectoparasites was likely similar in cavities and boxes, since we did not remove nest material between seasons and the colonization rate of ectoparasites in the study area was found to be very consistent across cavities by another researcher [59]. Thus, we hypothesize that nest predation risk and ectoparasitism rates likely did not differ between tree cavities and nest boxes in our study. We did not directly test the hypothesis that cleaning nest boxes influences fledging success but future work should compare uncleaned and cleaned boxes to determine how the practice of cleaning nest boxes interacts with ectoparasitsm and nest predation to influence fledging success.

Other investigators have found that older females tend to nest in boxes and have larger clutches than younger females that use tree cavities [3,13]. Some females were likely included twice in the study within and across nest types, but due to the sparsity of demographic data we were unable to account for this source of variation. In addition, we cannot rule out the possibility that females occupying the two nest types differed in terms of breeding age and/or experience. Our evidence supports the hypothesis that swallows preferred boxes perhaps because of their larger interior dimensions, and later-arriving and/or less competitive individuals (e.g., younger/less experienced individuals) were forced to nest in tree cavities. Thus it remains unknown how much of the difference in reproduction in boxes and cavities was due to the type of nest site versus the nature of birds using those nest sites. Further study is needed to examine how nest predation risk, predator types, nest box characteristics, and demographic variables might influence reproductive output in nest boxes versus tree cavities.

## Conclusions

Tree Swallows in North America depend on acquiring nesting cavities in ecological conditions that generally involve a limited supply of suitable tree cavities and a suite of strong nest site competitors. Our results that Tree Swallows laid larger, and more successful clutches in nest boxes suggests that nest boxes provide a reproductive advantage to Tree Swallow populations, particularly where competition for nest-sites is high. Our results contrast with earlier studies, which did not find greater fledging success in boxes relative to cavities [13,45]. If, as our findings suggest, nest types differ with birds nesting in boxes having higher fecundity, then regional or continental estimates of annual fecundity for Tree Swallows will be inflated when using data from nest boxes. It remains unclear why Tree Swallow populations in some parts of North America appear to be increasing while populations in other parts of the continent are in steady decline [1]. Other factors that could play a role in swallow population declines include changes in insect food supply resulting from historical or current pesticide application at breeding and/or wintering grounds [60,61], and habitat loss in breeding, wintering, or migration routes (see [2] for additional hypotheses). Further continent-wide research on how reproductive success is influenced by climate, microclimate in nest boxes and in tree cavities, and food availability is needed to understand the geographic variation in population changes of swallows and other aerial insectivores, and to determine the conditions where nest-box supplementation can favor increased reproductive output.
